# Clinical Assessment of the Effect of *Hypericum perforatum* Chewing Gum on Salivary *Streptococcus mutans* Count, pH, Plaque Index, and Gingival Bleeding: A Randomized Clinical Trial

**DOI:** 10.1002/cre2.70285

**Published:** 2026-01-09

**Authors:** Aylin Jamali, Hamed Hamishehkar, Mohammad Yousef Memar, Seyedeh Elham Mousavi Kalajahi, Maryam Kouhsoltani, Morteza Kosari‐Nasab

**Affiliations:** ^1^ Department of Oral and Maxillofacial Pathology, Faculty of Dentistry Tabriz University of Medical Sciences Tabriz Iran; ^2^ Drug Applied Research Center Tabriz University of Medical Sciences Tabriz Iran; ^3^ New Material and Green Chemistry Research Center Khazar University Baku Azerbaijan; ^4^ Infectious and Tropical Diseases Research Center Tabriz University of Medical Sciences Tabriz Iran; ^5^ Research Center, Nejati Industrial Group Tabriz Iran; ^6^ Department of Plant, Cell and Molecular Biology, Faculty of Natural Sciences University of Tabriz Tabriz Iran

**Keywords:** chewing gum, gingival bleeding, *Hypericum perforatum*, pH, *Streptococcus mutans*

## Abstract

**Objectives:**

*Hypericum perforatum L*. offers several beneficial effects, for example antioxidant, antitumor, wound healing. Our aim was to ascertain the effect of *H. perforatum* extract gum on salivary *Streptococcus mutans* count, pH, plaque index, and gingival bleeding.

**Materials and Methods:**

A total of 54 participants were assigned into two groups of gum with or without herbal extract. Unstimulated saliva was collected to assess *Streptococcus mutans* and pH.

**Results:**

Statistically significant differences in plaque index were observed between before and after use of gum in both groups (*p* < 0.01). There was a significant difference in plaque index after applying *H. perforatum* gum compared to placebo (*p* < 0.01). Statistically significant differences in gum bleeding between before and after use of herbal gum were observed (*p* < 0.01), but no significant difference was observed in gum bleeding between before and after gum use in placebo group (*p* = 0.24). A significant difference was observed in gum bleeding between herbal and placebo groups (*p* < 0.01). There was significant difference in *Streptococcus mutans* count between before and after gum use in both groups in two measurements (*p* < 0.01). Further, there was a significant difference in *Streptococcus mutans* count between herbal gum and placebo groups in two measurements (*p* < 0.01). Significant differences were observed in salivary pH between before and after consuming gum in herbal gum and placebo groups (*p* < 0.01 and *p* = 0.003, respectively). No significant difference in salivary pH was observed between two groups (*p* = 0.07).

**Conclusions:**

Our results revealed gum formulation of *H. perforatum* extract had significant effects on lowering salivary *Streptococci mutans* count, plaque index, and gingival bleeding compared to gum without extract group as well as their amounts before gum use. Although both gums elevated salivary pH, there was no statistically significant difference in short‐term measurements. Under the conditions of this study, *H. perforatum* gum can be considered a promising adjutant for oral health improvement.

## 1 Introduction

*St. John's Wort* (*Hypericum perforatum*) is native to Europe, Western Asia, and North Africa, and has been naturalized in the United States and Australia. This substance is recommended as a sedative as well as astringent agent, and has been traditionally used for treatment of wounds, irritability, neuralgia, bursitis, menopausal neurosis, anxiety, and depression. Today, it is widely employed in herbal products, homeopathic preparations, and flavoring ingredients (Gaster and Holroyd 2000; Müller 2003; Saddiqe et al. 2010). In addition, there is no doubt that this plant has antioxidant properties which is easily explained by numerous compounds of *H. perforatum* (Barnes et al. 2001; Süntar et al. 2016). Its antitumor activity has been shown in a study by Quiney et al. (2007) using several tests. *H. perforatum* extract contains several active compounds, including flavonoid derivatives, naphthodianthrones, and phloroglucinol (Halicioglu et al. 2015). *H. perforatum* extract contains six separate secondary metabolites, that is hyperforin, hypericin, hyperoside, quercetin, quercitrin, rutin. According to previous experiments, its quercetin and quercitrin metabolites are effective against *P. gingivalis* (responsible for gum disease) along with hyperforin and hypericin metabolites against *S. sabrinus*, *S. salivarius*, *S. sanguis mitis*, *Streptococcus mutans*, *A. actinomycetemcomitans,* and *C. albicans* (Agarwal and Chaudhary 2020; Balli et al. 2020; Beerhues 2006). Based on a study by Medina et al. (2006) in addition to these effects, the antiviral activity against herpes simplex viruses type 1 and type 2 has been noted. In a research by Chen et al. (2019), the antiviral activity of *H. perforatum* on infectious bronchitis virus (IBV) was evaluated in vitro and in vivo. This study provides evidence that the extract of *H. perforatum*, containing hyperoside, quercitrin, quercetin, sodium, and hypericin possesses anti‐IBV activity. Further, its anti‐IBV influence can be useful for developing new antiviral agents (Medina et al. 2006; Chen et al. 2019; Müller 2003). The findings of a study by Ipek Süntar and colleagues indicated that aqueous extract of *H. perforatum* is the most active fraction among other sub‐extracts against all tested oral pathogens namely *S. mutans*, *S. sobrinus*, *L. Plantarum*, and *E. faecalis*. Accordingly, they suggested that water‐soluble compounds of plant extracts are responsible for the antibacterial activity (Süntar et al. 2016).

The key question was whether the basic science knowledge of antibacterial, anti‐inflammatory, and antioxidant properties of *H. perforatum* could be effectively applied to a functional product. To the best of our knowledge, there was a lack of controlled, quantitative clinical evidence on the efficacy of gum formulation containing *H. perforatum* extract for ameliorating oral health indices, especially when compared to the control group (gum without extract group). This comparison is essential for isolating the specific impact of the herbal extract from the mechanical effects of chewing gum. Accordingly, this randomized, double‐blinded, controlled trial study aimed to compare the effects of chewing gum with and without *H. perforatum* extract on *S. mutans* count of saliva, salivary pH, plaque index, and gingival bleeding. The study was undertaken on 54 students along with staff of Dental Faculty with the participants assigned into two groups of chewing gum with or without *H. perforatum* extract. Unstimulated saliva was collected to ascertain *S. mutans* count of saliva and salivary pH before and after using the gums. The null hypothesis of this study was that the chewing gum containing *H. perforatum* extract would have no statistically significant influence on salivary *S. mutans* count, plaque index, gingival bleeding, and salivary pH compared to the control group (gum without the extract).

## 2 Methods

### 2.1 Study Design

**Figure 1:**
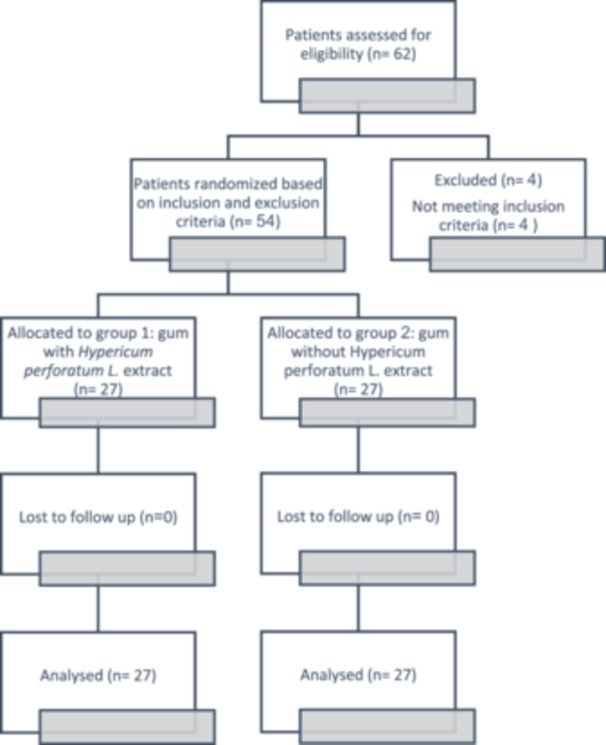
Consort flowchart of study.

This randomized, double‐blind, placebo‐controlled, parallel‐group clinical trial with the allocation ratio of 1:1 was performed at the Faculty of Dentistry, Tabriz University of Medical Sciences. This study was approved under an ethics code of IR.TBZMED.REC.1400.878 by ethics committee of Tabriz University of Medical Sciences and registered under the clinical trials code of IRCT20221021056257N1 at 2022‐01‐23. A total of 54 dental students and staff participated in this study. The consort flowchart of the study is displayed in Figure 1. All of the participants were informed verbally about the clinical process and informed written consents were obtained from all participants. They were also given the necessary assurance regarding the confidentiality of the received information. The study on confirmed individuals lasted from January 24, 2022 to April 18, 2022. The entry criteria for entering the study included: (1) No systemic diseases. (2) Acceptable oral hygiene (no obvious plaque or tartar). (3) Age range of 18–65 years. (4) Not to be pregnant or lactating or smoker. The exclusion criteria were: (1) Lack of appropriate cooperation. (2) Development of illness along the study period (Nagata et al. 2008; Wang et al. 2012; Kimbrough et al. 2002).

To determine the sample size, power and sample size software as well as the calculation formula of *n* = (*Z*_1−α/2_ + *Z*_1−β_)^2^ × (*s*_1_^2^ + *s*_2_^2^)/(*μ*_1_–*μ*_2_)^2^ (for studies testing two means of a trait) were utilized. The sample size was estimated according to the study by Abbasi and Haghgoo (2011). Considering *α* = 0.05, power of 80%, standard deviations of Irsha group (*s*1) = 0.268, and Persica group (*s*2) = 0.281, along with the means of group one (*μ*1) = 184.7 and group two (*μ*2) = 421.7 and an error of 10%, the number of estimated samples was determined 27 for each group.

The hygiene status of the participants was checked. They were divided into three categories according to their plaque index: (1) No plaque, (2) thin plaque on the edge of the cervical collar of the tooth, and (3) more plaque than group 2. The initial sampling was performed using convenience sampling method from a well‐defined target population. Importantly, after recruiting eligible volunteers, randomization was applied for group allocation. The method of randomization involved using a table of random numbers. Random numbers were assigned to one of the intervention or control groups, so the distribution of health status was identical between the two groups. Decayed, missing, and filled teeth index distribution was also the same between the two study groups (Wang et al. 2012; Kimbrough et al. 2002; Karami Nogourani et al. 2012). Since the study was double‐blind, except the supervisor as a third person in the study, none of the volunteers or individuals who undertook the project had any knowledge about the volunteers in the control or intervention groups. Also, to keep the information confidential, a code was defined for each volunteer.

## 3 Plant Materials and Extraction

### 3.1 Chewing Gum Samples' Preparation and Administration

The extraction was performed at the Pharmaceutical Laboratory, Drug Applied Research Center, Tabriz University of Medical Science. The flowering aerial parts of *H. perforatum* L. were collected from the botanic garden of Tabriz University of Medical Science and dried under shade at room temperature. This plant was identified and authenticated by Herbarium of Faculty of Pharmacy, Tabriz University of Medical Science (code: 4059 TBZFPH). After drying, the plant samples were ground into powder, whereby 2 kg of powders was extracted four times through maceration with 96% ethanol (4 L) for 48 h. The extract was concentrated in a rotary evaporator at a lowered pressure to yield 20 g of crude ethanolic extract. The dry extract was stored at −20°C before use, after which it was weighed and dissolved with 1% Tween 80 at the desired dose for experimental purposes.

Sugar‐free chewing gums containing 0.016% of *H. perforatum* extract and without plant extract were prepared. The amount of extract was adjusted based on the maximum taste tolerability (the extract was very bitter). The chewing gum samples were produced in Nejati industrial group. Accordingly, the gum base, which is a non‐nutritive, insoluble, and inert product, was heated at 50°C–60°C to get softer. Next, other compounds of the formulation were added to the gum base in certain amounts and mixed in a mixer equipped with temperature adjustment for uniformity and to reach a specific viscosity. The composition of chewing gum included mannitol (65%), gum base (23.7%), glycerin (5%), gum arabic (5%), fruit essence flavor and *H. perforatum* extract (1%), and lecithin as softener (0.3%). Nevertheless, sugar and glucose were omitted from the gum formulation, whereby hydrocolloids such as gum arabic were used along with the gum base for proper texture. After complete mixing, the gum paste was put into the extruder for proper shaping, after which it was cooled down in the refrigerator. Finally, the obtained chewing gum was cut into pieces of 5 g and packed in waxy paper followed by polyethylene. Each participant was trained to chew six pieces of gum every day, one piece after three meals and three pieces along the day for 14 days, with the chewing duration being 15 min for each piece. Each volunteer had to chew 84 gum pieces along the experiment. Over the study period, the subjects were asked not to alter their usual daily oral and dental hygiene. Eating or drinking except water was prohibited for 2 h before the visits. On the 1st and 15th days of the study, the volunteers were asked to eat in the early hours of the day and have a visit in Faculty of Dentistry to have their salivary *S. mutans* bacteria count and pH measured. Plaque and gingival bleeding were also recorded at the same session.

### 3.2 Antibacterial Activity Assay

An unstimulated salivary sample was collected from the participants for determining the *S. mutans* bacteria count before and after use of gums. The salivary samples were sent to the laboratory to determine the amounts of *S. mutans* bacteria. After collecting the samples, they were placed inside the transport environment and transferred to the Infectious Research Center of Sina Tabriz Hospital for assessment. The specific culture medium of Mitis salivarius agar was utilized to isolate the desired bacteria at a temperature of 37°. To prepare the culture medium 90.07 g of the substance was dispensed in 1000 mL of distilled water and heated to boil until completely dissolved in the environment. It was spread and sterilized using autoclave at 15 pounds pressure (121°C) for 15 min. It was then cooled to 50°C–55°C and 1 mL of sterile 1% potassium tellurite solution (FD052) was added. After adding the tellurite solution, the environment was not reheated. Finally, it was mixed well and poured into sterile petri plates.

Immediately after transferring the sample to the laboratory, it was mixed and diluted with saline solution to the concentrations of 1/10, 1/100, 1/1000, 1/100000, 1/1000000, and 1/10000000. It was spread on the surface of the culture medium in the form of grass (amount of 100 µL) and was cultured under the conditions of CO_2_ jar (10%) with colony counting carried out after 24–48 h. The steps were repeated twice. The plates in which the number of grown colonies were between 30 and 300 were counted (15). To confirm the morphology of bacteria, gram staining as well as catalase test were performed for all blue colored colonies. Warm blue color was utilized for gram‐positive chain cocci (short‐long). Catalase test was negative for all colonies (Nagata et al. 2008; Süntar et al. 2016).

### 3.3 pH Assessment

Salivary pH was measured immediately following unstimulated saliva collection using a calibrated pH meter (Hu‐Friedy, Metrohm, LLC, Switzerland) in order to minimize pH changes over time. The electrode was placed in the sample with the pH rounded to two decimal places. Specifically, 1 mL of saliva was measured by means of pH meter electrode at 0 min and 20 min after using chewing gum (Wang et al. 2012; Ballal et al. 2016). The final salivary pH value was the average of the two measurements (Wang et al. 2012; Kimbrough et al. 2002; Karami Nogourani et al. 2012).

### 3.4 Gingival Bleeding and Plaque Index Evaluation

The plaque index was calculated using O'Leary index. For this purpose, the patient's mouth was first rinsed with plain water and the surfaces of the teeth were tinted using a dye to detect dental plaques. The color change of different surfaces of each tooth was checked. For this purpose, each painted surface was examined for the accumulation of plaque exhibiting tablets at the junction of teeth and gums, where the stained areas were recorded with a red mark on the form. Ultimately, the number of discolored surfaces was divided by the number of teeth multiplied by four (or the total number of discolored surfaces) and expressed as a percentage. Gingival bleeding before and after the use of gum in both groups was ascertained using Williams periodontal probe (Hu‐Friedy, Michigan, LLC, USA) on four levels of the sulcus for 30 s (Nagata et al. 2008; Wang et al. 2012; Ziaie Rad and Taherian 2020).

### 3.5 Statistical Analysis

Statistical analyses included a series of descriptive analyses and analytical analyses. The data obtained from the study were described using statistical methods (mean ± standard deviation, frequency, and percentage). The data were analyzed by SPSS 26 and STATA 14.2 statistical software. For comparing the plaque index before and after the use of chewing gums in each of the groups and between two study groups, the beta regression test was employed. This test was also used to compare the amount of caries‐causing bacteria (*S. mutans*) in saliva. Gingival bleeding was compared before and after the use of chewing gums in each group and between two groups using generalized estimating equations analysis with Bonferroni correction. In order to compare pH of saliva in two groups and before and after using chewing gum in both groups, paired *t*‐test and covariance were utilized.

## 4 Results

A total of 54 dental faculty students along with staff participated in this study, 19 males and 35 females, ranging in age from 18 to 45 years. The age and gender distribution between two groups was matched. The numbers of participants who were randomly assigned, received treatment or excluded, and analyzed are outlined in Figure 1.

Table 1 reports plaque indices in *H. perforatum* chewing gum and gum without extract groups. The results of paired *t*‐test revealed a statistically significant difference in plaque indexes before and after the consumption of gum containing *H. perforatum* extract (*p*‐value < 0.001). The mean of plaque index before use of *H. perforatum* gum was almost 7% higher than after its use. Based on the results of the beta regression, a significant difference was observed before and after the use of placebo (*p*‐value < 0.001 and [5.16 and 4.06] 90%, CI = 4.61 B). A statistically significant difference was noted in the plaque index between the case and control groups; the mean of plaque index in *H. perforatum* gum group was almost 9% lower than that in the placebo (*p*‐value < 0.001).

**Table 1:** Plaque indexes of subjects consuming *Hypericum perforatum* chewing gum and gum without extract as control.

Study groups	Before use (mean ± standard deviation)	After use (mean ± standard deviation)	*p* value*	*p* value**
*H. perforatum*	25.60 ± 9.1815	18.04 ± 6.64	< 0.001	< 0.001
Gum without extract	30.36 ± 8.81	27.12 ± 7.64	< 0.001	

* Paired sample *t*‐test to compare before and after gum consumption.

** Beta regression to compare case and control groups.

Table 2 presents data on gingival bleeding. Using the analysis of generalized estimating equations with Bonferroni correction, a significant difference was found in gingival bleeding before and after the use of gum containing *H. perforatum* extract (*p*‐value < 0.001), but no significant difference was witnessed before and after use of placebo (*p*‐value = 0.24). Using the same test, a significant difference was noted in gingival bleeding between the case and control groups (*p*‐value < 0.001).

**Table 2:** Gingival bleedings of subjects using *Hypericum perforatum* chewing gum and gum without extract.

Study groups	Bleeding/not bleeding	Before use (mean ± standard deviation)	After use (mean ± standard deviation)	*p* value*	*p* value**
*H. perforatum*	Bleeding on probing	5 (20%)	4 (16%)	< 0.001	< 0.001
	No Bleeding	20 (80%)	21 (84%)		
Gum without extract	Bleeding on probing	3 (12%)	3 (12%)	0.24	
	No Bleeding	22 (88%)	22 (88%)		

* Generalize estimating equations (GEE) test to compare before and after gum consumption.

** GEE test to compare case and control groups.

Based on the analysis of generalized estimating equations with Bonferroni correction, statistically significant differences were observed in caries‐causing bacteria (*S. mutans*) count in saliva before and after the use of *H. perforatum gum* (*p*‐value < 0.001) and gum without extract (*p*‐value < 0.001) in two measurements. Further, significant differences were observed between *H. perforatum* and placebo groups in two measurements (*p*‐value < 0.001). Table 3 presents *S. mutans* counts in study groups before and after use of chewing gums in study groups.

**Table 3:** *Streptococcus mutans* count in saliva of *Hypericum perforatum* gum and gum without extract consumers.

Study groups	Measurements	Before use (mean ± standard deviation)	After use (mean ± standard deviation)	*p* value	*p* value*	*p* value**
*H. perforatum*	1st measurement	498,148 ± 165,669	107,983 ± 85,188	< 0.001	< 0.001	< 0.001
	2nd measurement	504,814 ± 186,039	120,943 ± 95,188	< 0.001		
Gum without extract	1st measurement	597,407 ± 129,335	498,592 ± 198,884	< 0.001	< 0.001	
	2nd measurement	481,148 ± 160,022	505,925 ± 193,753	< 0.001		

* GEE test to compare before and after gum consumption.

** GEE test to compare case and control groups.

Table 4 details the pH of saliva in the study groups. Using paired *t*‐test, a statistically significant difference was observed in salivary pH before and after the consumption of chewing gum containing *H. perforatum* extract (*p*‐value < 0.001). Also, a significant difference was noted in salivary pH before and after use of placebo (*p*‐value = 0.003). Using covariance analysis, no significant difference in salivary pH was found between *H. perforatum* gum and gum without extract consumers (*p*‐value = 0.07).

**Table 4:** Salivary pH in subjects consuming *Hypericum perforatum* chewing gum and gum without extract.

Study groups	Before use (mean ± standard deviation)	After use (mean ± standard deviation)	*p*‐value*	*p*‐value**
*H. perforatum*	6.37 ± 0.40	6.65 ± 0.43	< 0.001	0.07
Gum without extract	6.57 ± 0.52	6.60 ± 0.51	0.003	

* Paired sample *t*‐test to compare before and after gum consumption.

** ANCOVA to compare case and control groups.

Table 5 indicates that the chance of no bleeding in the *H. perforatum* chewing gum group has been 38% lower than the gum without extract group (OR = 0.719, CI%95: [0.145, 3.569]).

**Table 5:** Risk estimate for bleeding.

Study groups	Value	95% Confidence interval	
		Lower	Upper
Odds ratio for code bleeding (0.00/1.00)	0.719	0.145	3.569
*H. perforatum*	0.856	0.423	1.733
Gum without extract	1.191	0.484	2.931
Number of valid cases	54		

## 5 Discussion

This study explored the influence of *H. perforatum* extract in herbal chewing gum on reducing the amount of *S. mutans* bacteria in saliva. There was a significant difference in the amount of caries‐causing bacteria (*S. mutans*) before and after the use of *H. perforatum* gum and placebo, with a significant difference also observed between the *H. perforatum* gum and the placebo group, reflecting the antibacterial properties. These results are supported by a study done by Balli et al. (2020). In another study by Schempp et al. (1999) the results revealed that hyperforin was effective on Gram‐positive and Gram‐negative microorganisms where different concentrations of hyperforin (0.1, 1, 10, and 100 μg/mL) were effective on all Gram‐positive microorganisms. In a study by Reichling and his colleagues (2001), the impact of the organic extract of the aerial parts of *Hypericum perforatum L*. was reported against Gram‐positive bacteria, including active methicillin‐resistant *Staphylococcus aureus*. Cecchini and colleagues (2007) inspected the antimicrobial effects of several plants, including extract of *H. perforatum*, which demonstrated the effect of the extract of this plant on *S. aureus* bacteria. Hübner (2003) proposed the potential use of hyperforin as an antibiotic in a clinical study in which no resistance to hyperforin was observed at low concentrations of hyperforin even in strains less sensitive to it.

The results obtained from our research can be confirmed according to the mentioned studies on the *H. perforatum* plant and the influence of this substance on different types of bacteria.

Plaque index is an established indicator for determining the level of personal hygiene, whereby the absence of gum bleeding during probing indicates the absence of active inflammation and periodontal disease. In this study, owing to the good oral and dental hygiene of the majority of the studied population, no active periodontal disease was observed, but in a small percentage of the studied subjects, bleeding occurred 30 s after the probing. There was a statistically significant difference in the extent of bleeding before and after using the gum in both groups. There was also a statistically significant difference between case and control groups. The analysis of percentage of plaque index revealed the effect of *H. perforatum* on lowering plaque with its effect on salivary flow and the antibacterial effect of this extract in the group of users of chewing gum containing plant extract compared to the control group.

In a study by Halicioglu et al. (2015) the impact of systemic *St. John's wort* (*Hypericum perforatum*) yeast was investigated on new bone formation in the maxillary suture in rats, whereby it was observed that it prevents relapse and inflammation. In another study, the application of mouthwash containing *H. perforatum* extract significantly reduced plaque amount and gingivitis compared to saline as a mouthwash (Nisha et al. 2021). This is in accordance with the findings of our study and emphasize the effect of this extract on lowering inflammation and plaque accumulation. A 3‐week study was conducted in the Netherlands, which confirmed the positive influence of gum containing artificial sweeteners compared to gum base on gingivitis and the amount of plaque (Keukenmeester et al. 2014). This study justified the reduction of plaque in placebo group, owing to increase in salivary flow and its antiseptic effect. Consuming chewing gum containing xylitol 3–6 times a day after meals with the rationale of reducing the number of bacteria, strengthening remineralization, increasing salivary flow, and reducing acid production was recommended. Chewing gum, turpentine, and topical solutions containing CPP‐ACP can mineralize white spots. It has been found that regular use of CPP‐ACP is effective in enamel remineralization (Ritter et al. 2019).

The pH level in the mouth can affect the health of teeth and gums; and adjusting the pH level reduces oral bacteria, thus mitigating the risk of gum disease and tooth decay. Saliva has specific buffering mechanisms, for example bicarbonate, phosphate, and some protein systems that not only have buffering effects but also provide ideal conditions to eliminate certain bacterial compounds that require low pH level for survival. Chewing gum by promoting the flow of saliva reduces the residual food and microorganisms in the oral cavity and boosts the buffering capacity. Eating sugar‐free gum is a great way to elevate salivary pH and flow rate. Previous studies have indicated that sugar‐free chewing gums reduce dental plaque and salivary acid production, and enhance saliva secretion (Polland et al. 2003). In this study, there was a significant difference in the pH level of saliva before and after consuming *H. perforatum* gum. However, considering the effect of the control group's chewing gum which did not contain sugar on the pH of saliva, there was no significant difference between the control and case groups.

The null hypothesis of the study was chewing gum containing *H. perforatum* extract would have no statistically significant effect on salivary *S. mutans* count, plaque index, gingival bleeding, and salivary pH compared to the control group (gum without extract). The study results (e.g., *p*‐value < 0.001 for the difference in bacterial count and plaque index between the two groups) reject this null hypothesis in favor of the alternative hypothesis (H₁), indicating existence of a significant difference.

Considering the limitation of the present study, further investigations with larger sample sizes, longer follow‐up periods, as well as different concentrations of the extract are recommended to confirm these results and optimize the formulation. Although no adverse events were reported in our short‐term trial, *H. perforatum* is a known photosensitizing agent when used systemically owing to its hypericin content (Balli et al. 2020; Beerhues 2006). While the risk of systemic photosensitivity from a topical oral product is far lower because of minimal systemic absorption, the potential for localized phototoxic reactions cannot be entirely ruled out. There is also a potential of allergic reaction to any of the several compounds in the extract. Future studies with longer follow‐up periods should monitor for signs of mucosal irritation, burning sensations, or ulceration.

## 6 Conclusion

Within the limitations of this controlled trial study, the gum formulation of *H. perforatum* extract had significant effects on lowering salivary *S. mutans* count, plaque index and gingival bleeding compared to gum without extract group as well as their amounts before gum use. According to the beneficial properties of *H. perforatum* in lessening the amount of Gram‐positive bacteria, the effect of this plant extract on *S. mutans* can be justified. Further, by lowering the pathogenic bacteria, secondary acid metabolites are reduced which enhances the buffering properties of saliva potentially in the long term. Thanks to the antimicrobial and anti‐inflammatory properties of this plant, plaque control and hence plaque index can be improved. By promoting plaque control and reducing inflammation, reducing gingival bleeding is acceptable. Although both gums elevated the salivary pH, there was no statistically significant difference in short‐term measurements. Under the conditions of this study, *H. perforatum* gum can be considered a promising adjutant for oral health improvements.

## Author Contributions

Conceptualization: Hamed Hamishehkar, Maryam Kouhsoltani, and Morteza Kosari‐Nasab. Data curation: Aylin Jamali and Seyedeh Elham Mousavi Kalajahi. Formal analysis: Hamed Hamishehkar, Mohammad Yousef Memar, Maryam Kouhsoltani, and Morteza Kosari‐Nasab. Funding Acquisition: Maryam Kouhsoltani. Investigation: Aylin Jamali and Seyedeh Elham Mousavi Kalajahi. Methodology: Hamed Hamishehkar, Mohammad Yousef Memar, Seyedeh Elham Mousavi Kalajahi, Maryam Kouhsoltani, and Morteza Kosari‐Nasab. Project administration: Aylin Jamali, Mohammad Yousef Memar, and Seyedeh Elham Mousavi Kalajahi. Software: Maryam Kouhsoltani, Hamed Hamishehkar, and Morteza Kosari‐Nasab. Supervision: Hamed Hamishehkar, Mohammad Yousef Memar, Maryam Kouhsoltani, and Morteza Kosari‐Nasab. Validation: Hamed Hamishehkar, Mohammad Yousef Memar, Maryam Kouhsoltani, and Morteza Kosari‐Nasab. Visualization: Hamed Hamishehkar, Mohammad Yousef Memar, Maryam Kouhsoltani, and Morteza Kosari‐Nasa. Writing – original draft: Aylin Jamali and Maryam Kouhsoltani. Writing – review and editing: Hamed Hamishehkar, Mohammad Yousef Memar, Seyedeh Elham Mousavi Kalajahi, Maryam Kouhsoltani, and Morteza Kosari‐Nasab.

## Conflicts of Interest

The authors declare no conflicts of interest.

## Data Availability

The data that support the findings of this study are available from the corresponding author upon reasonable request.
